# *APOE* genotype influences insulin resistance, apolipoprotein CII and CIII according to plasma fatty acid profile in the Metabolic Syndrome

**DOI:** 10.1038/s41598-017-05802-2

**Published:** 2017-07-24

**Authors:** Rosalind Fallaize, Andrew L. Carvalho-Wells, Audrey C. Tierney, Carmen Marin, Beata Kieć-Wilk, Aldona Dembińska-Kieć, Christian A. Drevon, Catherine DeFoort, José Lopez-Miranda, Ulf Risérus, Wim H. Saris, Ellen E. Blaak, Helen M. Roche, Julie A. Lovegrove

**Affiliations:** 1 Hugh Sinclair Unit of Human Nutrition and Institute for Cardiovascular and Metabolic Research, University of Reading, Whiteknights Reading, RG6 6AP UK; 2 School of Life and Medical Sciences, University of Hertfordshire, College Lane Hatfield, AL10 9AB UK; 3 Nutrigenomics Research Group, University College Dublin Conway Institute, University College Dublin, Dublin, Ireland; 4 Lipids and Atherosclerosis Unit. Instituto Maimónides de Investigación Biomédica de Córdoba (IMIBIC), Reina Sofia University Hospital, University of Córdoba, Córdoba, Spain; 5 Department of Metabolic Diseases, University Medical College, Krakow, Poland; 6 Department of Clinical Biochemistry, Jagiellonian University Collegium Medicum, Kraków, Poland; 7 Department of Nutrition, Institute of Basic Medical Sciences, Faculty of Medicine, University of Oslo, Oslo, Norway; 8 INSERM, 476 Human Nutrition and Lipids, Marseille, France; 9 Department of Public Health and Caring Sciences/Clinical Nutrition and Metabolism, Uppsala University, Uppsala, Sweden; 10 Department of Human Biology, NUTRIM School for Nutrition and Translational Research in Metabolism, Maastricht University Medical Centre+ (MUMC+) Maastricht, Maastricht, The Netherlands

## Abstract

Metabolic markers associated with the Metabolic Syndrome (MetS) may be affected by interactions between the *APOE* genotype and plasma fatty acids (FA). In this study, we explored FA-gene interactions between the missense *APOE* polymorphisms and FA status on metabolic markers in MetS. Plasma FA, blood pressure, insulin sensitivity and lipid concentrations were determined at baseline and following a 12-week randomized, controlled, parallel, dietary FA intervention in 442 adults with MetS (LIPGENE study). FA-*APOE* gene interactions at baseline and following change in plasma FA were assessed using adjusted general linear models. At baseline *E4* carriers had higher plasma concentrations of total cholesterol (TC), low-density lipoprotein cholesterol (LDL-C) and apolipoprotein B (apo B) compared with *E2* carriers; and higher TC, LDL-C and apo B compared with *E3/E3*. Whilst elevated plasma n-3 polyunsaturated FA (PUFA) was associated with a beneficially lower concentration of apo CIII in *E2* carriers, a high proportion of plasma C16:0 was associated with insulin resistance in *E4* carriers. Following FA intervention, a reduction in plasma long-chain n-3 PUFA was associated with a reduction in apo CII concentration in *E2* carriers. Our novel data suggest that individuals with MetS may benefit from personalized dietary interventions based on *APOE* genotype.

## Introduction

The Metabolic Syndrome (MetS) is characterized by a clustering of risk factors related to cardiovascular disease (CVD) and type-II diabetes (T2D), including abdominal obesity, insulin resistance, hypertension, dyslipidemia and inflammation^1, 2^. Individuals with 4–5 features of MetS have a 3.7-fold increased risk of CVD and 24-fold increased risk of T2D^3^. The primary goal of clinical management is to reduce risk for metabolic and atherosclerotic disease^4^. This is achieved by targeting modifiable risk factors such as obesity, physical inactivity and inappropriate diets, in addition to smoking. Unhealthy diets, such as those high in saturated fatty acids (SFA), have been shown to increase low-density lipoprotein cholesterol (LDL-C) and CVD risk^5^. Whilst a large scale meta-analysis (n = 347,747) conducted in 2010 revealed no association between SFA and risk of stroke or CVD^6^, recent Cochrane review found a beneficial impact of SFA reduction on CVD risk^7^. Nevertheless, a reduction in LDL-C and CVD risk has been observed following replacement of SFA with unsaturated FA^8–10^, with evidence for a greater benefit of polyunsaturated FA (PUFA)^7, 11^. Given that responsiveness to dietary fat alteration is highly heterogeneous, there is interest in the impact of non-modifiable risk factors, such as genetics, to assist in prevention and treatment of chronic diseases such as MetS.

Of particular interest is the apolipoprotein E (*APOE*) genotype, a key regulator of lipoprotein metabolism, shown to account for up to 7% of the variance observed in total cholesterol (TC) and low-density lipoprotein cholesterol (LDL-C)^12^. Polymorphisms in the *APOE* gene, rs429358 (Cys112Arg) and rs7412 (Arg158Cys), encode three common alleles, ε2 (Cys122 and Cys158), ε3 (Cys112 and Arg158) and ε4 (Arg112 and Arg158), which combine to form 6 genotypes, ε2/ε2, ε2/ε3, ε2/ε4, ε3/ε3, ε3/ε4 and ε4/ε4. Some scientists have reported a higher incidence of the ε4 allele among MetS subjects^13, 14^. The ε4 allele has been associated with increased TC, LDL-C, coronary artery disease (CAD) mortality and reduced high-density lipoprotein cholesterol (HDL-C) concentrations^12, 15–19^. However, associations between ε4 and CAD mortality in the 2007 meta-analysis (n = 47,467) were moderate (OR 1.06, 95% CI 0.99–1.13)^16^. The *APOE* genotype has also been associated with the development of Alzheimer’s^20^. To date, intervention studies have suggested that *E4* carriers may be more sensitive to dietary cholesterol, total fat, SFA and long chain n-3 PUFA (LC n-3 PUFA, comprising EPA and DHA) modulation^21–26^. Thus, it has been suggested that the detrimental effects of the *APOE* genotype might be ameliorated by modulating the type and quantity of dietary fat^27^. More recently, independent associations between the ε*4* allele and CVD risk have been observed among individuals with MetS^28^.

Although there has been a wealth of interest in the functional impact of polymorphisms at the *APOE* locus, a limited number of RCT have investigated dietary fat manipulation and *APOE* genotype, and very few in subjects with the MetS phenotype. Thus, we examined the relationship between the missense *APOE* polymorphism, habitual FA status and following dietary FA intervention on metabolic markers in a MetS population.

## Subjects and Methods

The LIPGENE parallel dietary intervention study was conducted at 8 EU centers: Dublin, Ireland; Reading, UK; Oslo, Norway; Marseille, France; Maastricht, The Netherlands; Cordoba, Spain; Krakow, Poland; and Uppsala, Sweden, as described previously^29^. The study was conducted according to the Declaration of Helsinki and registered with the US National Library of Medicine ClinicalTrials.gov registry (NCT00429195; 01/30/2007). Ethical approval for the study was granted at each center and informed written consent was obtained from each subject prior to participation.

### Participants

A total of 442 of the 486 participants randomized into the LIPGENE study were included in the present analysis owing to missing genotype data for *APOE* polymorphism in 44 participants. The group consisted of 248 women and 194 men, with a mean (±SEM) age of 54 (±1) years. The inclusion criteria were age 35–70 years, BMI 20–40 kg/m^2^ and presence of the MetS, as defined by a modified version of the NCEP ATP III criteria, in which participants required at least three of the following: fasting plasma glucose 5·5–7.0 mmol/l, serum triacylglycerol (TAG) ≥ 1·5 mmol/l, serum HDL-cholesterol <1·0 mmol/l in males and <1·3 mmol/l in females, waist circumference >102 cm in males and >88 cm in females, and elevated blood pressure (BP) (systolic BP ≥ 130 mmHg, diastolic BP ≥ 85 mmHg or on prescribed BP-lowering medication).

### Study design

Participants recruited to the 12-week study were randomized according to age, sex and fasting plasma glucose using Minimization Programme for Allocating Participants to Clinical Trials (Department of Clinical Epidemiology, The London Hospital Medical College, UK) to one of four isoenergetic diets that differed according to fat quantity and quality: high fat SFA-rich diet, high fat monounsaturated fatty acids (MUFA)-rich diet, low-fat high-complex carbohydrate diet supplemented with 1.24 g/d LC n-3 PUFA (Marinol C-38) or low-fat high-complex carbohydrate diets supplemented with 1 g/d high-oleic acid sunflower oil^29^. Approximately 120 subjects were assigned to each dietary group. Dietary targets were obtained using a novel dietary exchange model previously described^30^. Habitual assessment of dietary intake, using a 3-day weighed food record, formed the basis of the isoenergetic (<0.2 kg weight change) dietary fat modification. Dietary change was facilitated via detailed dietetic consultation and re-assessed at weeks 6 and 12 using a 3-day weighed food record to ensure dietary compliance. Prior to the intervention, participants were required to complete a health and lifestyle questionnaire to assess habitual physical activity, smoking status, alcohol intake and socio-demographic status, which remained consistent throughout the study. Anthropometric measures, blood pressure and biological samples were taken with consent, following a 12-hour overnight fast before and after the 12-week intervention. Collection of samples was conducted according to standardized operating procedures to ensure consistency across centers. Detailed recruitment and study procedures have been published previously^29^.

### Biochemical analysis

Plasma TC, HDL-cholesterol, LDL-cholesterol, TAG, non-esterified fatty acids (NEFA) and glucose concentrations were analyzed using enzymatic colorimetric methods (Instrumentation Laboratory, Warrington, UK; WAKO NEFA C kit, Alpha Laboratories, Hampshire, UK). Assays were used for quantification of plasma concentrations of apolipoproteins AI, B and E (Behring Werke AG, Marburg, Germany), triacylglycerol-rich lipoprotein (TRL) apo B48, and apo CIII and apo CII (Diasys, Bouffe´mont, France). Plasma FA were extracted and transmethylated with borontrifluoride in methanol and fatty acid methyl esters (FAME) of plasma fatty acids analysed using a Shimadzu GC-14A gas liquid chromatograph fitted with a Shimadzu C-r6A integrator and a 25 M BP 21 polar aluminium silica column (Shimadzu, Japan). Fatty acids were identified using FAME standards (Sigma-Aldrich Company Ltd, Dorset, England); see ref. 31 for detailed description of method. Enzyme-linked immunosorbent assay (ELISA) was used to determine C-reactive protein (CRP) (BioCheck Inc., Foster City, CA, USA). Serum insulin was determined by solid-phase, two-site fluoro-immunometric assay (Wallac Oy, Turku, Finland). Homeostasis model assessment (HOMA-IR) was calculated as: [(fasting plasma glucose × fasting plasma insulin)/22.5]^32^. All samples were analyzed centrally.

### DNA extraction and genotyping

DNA was extracted from the buffy coat of whole fasted blood using the AutoPure LS automated system (Gentra Systems Inc, Minneapolis, MN). Samples with low-yield (<10 ng) were subjected to whole-gene amplification using the REPLI-g kit (Qiagen Ltd, West Sussex, UK). The SNP rs7412 and rs429358 were genotyped according to LIPGENE protocol by Illumina Inc. (San Diego, CA), with use of the Golden Gate Assay on a BeadStation 500 G system (Illumina Inc, San Diego, CA). Adherence to Hardy–Weinberg equilibrium at each SNP locus was determined using the chi^2^ test with 1 degree of freedom; SNPs were in accordance with the Hardy–Weinberg equilibrium.

### Statistical analysis

Data are expressed as means ± SEM. All data were checked for skewness and kurtosis and where necessary normalized by log (WC, NEFA, HDL-C, TC-HDL-C ratio, TAG, apo CIII, glucose, insulin, HOMAIR and CRP) and square root (apo E and apo CII) transformation. Associations between *APOE* genotype and biomarkers were determined using a general linear model (GLM) with adjustment for age, sex, center and BMI. Where a significant overall genotype effect was observed (*P* ≤ 0.05) a post hoc test (Bonferroni) was applied to determine between genotype differences.

Nutrient-gene interactions were determined using the adjusted GLM (as described) but with the addition of a genotype × fatty acid (% of total plasma lipids) interaction term (see Supplementary Table 1 for list of FA evaluated). Where a significant nutrient-gene interaction was found with the FA evaluated as a continuous variable, the result was verified by dichotomizing the dataset by median plasma FA concentrations (as a categorical variable) to compare the effect of genotype within subjects who had similar habitual diets, e.g. within the higher intake (above median) or lower intake (below median) groups, using a post hoc test (Bonferroni).

The interactions between genotype and plasma FA (SFA, total PUFA and LC n-3 PUFA) on each biochemical variable, following dietary intervention, was assessed by using 0% change in plasma FA (% of total plasma lipids) to dichotomize subjects, and then using the resulting groups as fixed factors in a GLM (i.e. reduction in plasma FA and increase in plasma FA). This method provides an objective assessment of change in nutrient intake, irrespective of errors in dietary reporting and study compliance. Furthermore, splitting the data according to diet allocation (i.e. divided into four) would have led to insufficient carrier numbers in each group. Using change in plasma FA more clearly distinguishes the impact of actual dietary change on outcome variables. The plasma FA examined were those manipulated in the LIPGENE intervention: SFA, MUFA and LC n-3 PUFA. Non-parametric data were transformed using log (Y + *a*), where *a* is the minimum constant (triglyceride-rich lipoprotein cholesterol fraction (TRL-C), apo A1, apo CII, apo CIII, apo E, glucose and CRP)^33^. As previously, the interaction term genotype × FA was added to a GLM, with the biochemical variable as the response variable and the respective pre-intervention variable as a covariate. Additional covariates added to the model were age, sex, center and weight change [post intervention weight (kg) – pre intervention weight (kg)]. Where a significant overall genotype effect was observed (*P* ≤ 0.05) a post hoc test (Bonferroni) was applied to determine between and within group differences. Statistical analysis was performed using Minitab for Windows (version 16, Coventry, UK). Post-hoc power analyses were conducted using G*Power^34^.

## Results

Genotypes were reported as *E2* carriers, comprising (*E2/E2* n = 3, and *E2/E3*, n = 43), *E4* carriers (*E4/E4* n = 12 and *E4/E3* n = 103), homozygous *E3/E3* (n = 264) and *E2/E4* carriers (n = 17).

### Genotype frequency

Genotype and allele frequency according to LIPGENE center are shown in Table 1. A geographic cline can be seen with respect to allele frequency, with the most southerly country expressing the lowest frequency of the ε4 allele and the most northerly country greater than 2-fold higher (*E3/E4* and *E4/E4* combined frequency, 8.6% for Cordoba, Spain versus 22.8% for Oslo, Norway).

**Table 1:** Frequency of *APOE* genotype by LIPGENE Dietary Fatty Acid Intervention Study center (n = 442).

	All	Norway	Sweden	Ireland	Netherlands	UK	Poland	France	Spain
**Genotype (n, %)**									
*E2/E2*	3 (0.7)	0 (0.0)	0 (0.0)	0 (0.0)	1 (2.2)	1 (1.7)	0 (0.0)	0 (0.0)	1 (1.4)
*E2/E3*	43 (9.7)	5 (8.7)	4 (8.3)	7 (12.0)	7 (15.9)	5 (8.6)	7 (10.3)	2 (5.1)	6 (8.5)
*E2/E4*	17 (3.8)	3 (5.2)	3 (6.2)	1 (1.7)	1 (2.2)	2 (3.4)	2 (2.9)	2 (5.1)	3 (4.2)
*E3/E3*	264 (59.7)	27 (47.3)	26 (54.1)	37 (63.8)	26 (59.0)	26 (44.8)	42 (61.8)	28 (71.8)	52 (74.2)
*E3/E4*	103 (23.3)	21 (36.8)	15 (31.2)	10 (17.2)	7 (15.9)	21 (36.2)	16 (23.5)	6 (15.3)	7 (10.0)
*E4/E4*	12 (2.7)	1 (1.7)	0 (0.0)	3 (5.1)	2 (4.5)	3 (5.1)	1 (1.4)	1 (2.5)	1 (1.4)
*E2* carriers^a^	46 (10.4)	5 (8.7)	4 (8.3)	7 (12.0)	8 (18.1)	6 (10.3)	7 (10.2)	2 (5.1)	7 (10.0)
*E4* carriers^b^	115 (26.0)	22 (38.6)	15 (31.2)	13 (22.4)	9 (20.4)	24 (41.3)	17 (24.9)	7 (17.9)	8 (11.4)
ALL	442	57	48	58	44	58	68	39	70
**Allele frequency (%)**									
ε2	7.5	7.0	7.3	6.9	11.4	7.8	6.6	6.3	7.9
ε3	76.2	70.2	74.0	78.4	75.0	67.2	78.8	81.3	83.6
ε4	16.3	22.8	18.8	14.7	13.6	25.0	14.7	12.6	8.6

Values are n (%). ^a^Genotype groups combined; E2 carriers represent E2/E2 and E2/E3. ^b^Genotype groups combined; E4 carriers represent E4/3 and E4/E4.

### Subject characteristics

In Table 2, subject anthropometry and fasted lipid profiles are reported according to *APOE* genotype. *E2* carriers had significantly lower baseline plasma concentrations of TC, LDL-C and apo B when compared with the *E4* carriers; and lower TC, LDL-C and apo B compared to the *E3/E3* group. TC:HDL-C ratio was significantly higher in *E4* carriers compared with *E2* carriers and apo E in *E2* carriers (compared with *E3/E3* and E4 carriers). Apo E was also significantly higher in *E2/E4* carriers when compared with *E4* carriers. In addition, *E4* carriers had significantly higher TRL-C and lower CRP than *E3/E3*. There was no evidence of a genotype-dependant difference in baseline anthropometry, blood pressure or markers of insulin resistance. Plasma FA values (% total plasma FA) and dietary fat intakes (g) according to APOE genotype are reported in Supplementary Tables 2 and 3; no significant differences according to genotype were observed. E2/E4 carriers were removed from subsequent analysis due to their low population frequency.

**Table 2:** Effect of the *APOE* genotype on anthropometric variables, blood pressure, fasted plasma- and serum- profiles in the LIPGENE Dietary Fatty Acid Intervention Study (*n* = 442; males *n* = 194, females *n* = 248).

	All (n = 442)	*E2* carriers (n = 46)	*E3/E3* (n = 264)	*E4* carriers (n = 115)	*E2/E4* (n = 17)	P
Age (y)	55 ± 1	56 ± 1	54 ± 1	55 ± 1	53 ± 2	0.026
BMI (kg/m^2^)	31.6 ± 1.0	32.6 ± 0.7	32.6 ± 0.3	32.4 ± 0.5	31.6 ± 1.1	0.316
Waist (cm)	104 ± 3	107 ± 2	106 ± 1	105 ± 1	104 ± 2	0.894
Diastolic BP (mm Hg)	86 ± 2	87 ± 1	86 ± 1	86 ± 1	87 ± 2	0.729
Systolic BP (mm Hg)	137 ± 4	137 ± 2	139 ± 1	140 ± 1	137 ± 4	0.744
TC (mmol/L)	5.15 ± 0.69	4.98 ± 0.16^a^	5.38 ± 0.06^b^	5.53 ± 0.09^b^	5.03 ± 0.21	0.015
LDL-C (mmol/L)	3.08 ± 0.11	2.67 ± 0.16^a^	3.27 ± 0.06^b^	3.57 ± 0.09^b^	3.14 ± 0.18	0.001
NEFA (µmol/L)	610 ± 27	675 ± 37	597 ± 14	641 ± 23	593 ± 58	0.129
HDL-C (mmol/L)	1.08 ± 0.04	1.13 ± 0.05	1.11 ± 0.02	1.08 ± 0.02	1.14 ± 0.09	0.234
TC:HDL-C ratio	5.12 ± 0.20	4.65 ± 0.21^a^	5.05 ± 0.07	5.38 ± 0.14^b^	4.92 ± 0.49	0.035
TRL-C (mmol/L)	1.82 ± 0.13	0.46 ± 0.05	0.34 ± 0.01^a^	0.45 ± 0.04^b^	0.47 ± 0.10	0.493
TAG (mmol/L)	0.47 ± 0.05	1.91 ± 0.16	1.71 ± 0.05	1.94 ± 0.10	1.86 ± 0.18	0.371
TRL-TAG (mmol/L)	0.93 ± 0.09	0.89 ± 0.09	0.82 ± 0.03	0.98 ± 0.07	0.89 ± 0.15	0.286
Apo AI (mg/L)	1.37 ± 0.03	1.43 ± 0.04	1.41 ± 0.02	1.38 ± 0.02	1.42 ± 0.07	0.590
Apo B (mg/L)	0.95 ± 0.02	0.88 ± 0.04^a^	1.03 ± 0.01^b^	1.09 ± 0.02^b^	0.90 ± 0.04	< 0.001
Apo B48 (mg/L)	0.85 ± 0.13	0.67 ± 0.10	0.79 ± 0.06	0.78 ± 0.09	1.25 ± 0.33	0.197
TRL apo B (mg/L)	56.7 ± 6.1	51.1 ± 7.3	44.9 ± 2.4	59.5 ± 5.4	60.6 ± 11.7	0.283
Apo CII (mg/L)	46.75 ± 2.2	49.0 ± 3.0	44.7 ± 1.0	45.7 ± 1.8	52.1 ± 4.6	0.353
Apo CIII (mg/L)	152 ± 7	169 ± 10	157 ± 3	159 ± 5	159 ± 12	0.777
Apo E (mg/L)	43.8 ± 3.11	55.1 ± 4.64^a,c^	39.9 ± 0.73^b,c^	38.0 ± 1.37^b^	50.4 ± 1.98^c^	< 0.001
Glucose (mmol/L)	5.92 ± 0.21	6.04 ± 0.11	5.91 ± 0.06	6.10 ± 0.12	5.92 ± 0.25	0.113
Insulin (µIU/mL)	9.0 ± 1.5	9.7 ± 0.9	10.1 ± 0.4	10.4 ± 0.6	9.0 ± 1.5	0.996
HOMA-IR	2.51 ± 0.56	2.64 ± 0.25	2.65 ± 0.10	2.87 ± 0.19	2.51 ± 0.57	0.892
CRP (mg/L)	5.18 ± 0.20	5.32 ± 0.77	5.51 ± 0.24^a^	4.48 ± 0.41^b^	4.45 ± 0.41	0.028

Abbreviations: BMI, body mass index; SBP, systolic blood pressure; DBP, diastolic blood pressure; TC, total cholesterol; LDL-C, low-density lipoprotein cholesterol; HDL-C, high-density lipoprotein cholesterol; NEFA, non-esterified fatty acid; TAG, triacylglycerol; TRL-C, triglyceride rich lipoprotein cholesterol fraction; TRL-TG, triglyceride rich lipoprotein triglyceride fraction; CRP, C-reactive protein. Values are means ± s.e.m. Models were adjusted for center, gender, age and BMI. Where *P* for genotype <0.05, a post-hoc Bonferroni test were used to determine a between group effect. Superscript letters ^a^ and ^b^ denote significant differences in means (P < 0.05).

### Habitual plasma FA and genotype interactions at baseline

Significant gene-nutrient interactions between *APOE* and plasma SFA (C14:0, C16:0, C18:0), MUFA (C16:1, C18:1, C20:1), n-3 PUFA (C18:4, C20:5, C22:5, C22:6) and n-6 PUFA (C18:2, C18:3, C20:3, C20:4 and C22:4), evaluated as a continuous variable (as a % of total FA), on fasting metabolic biomarkers are reported in Table 3. Where a significant interaction was observed, data were dichotomized by median to give categorical data: total SFA (31.1%), C14:0 (1.76%), C16:0 (25.7%), C18:0 (4.1%), total MUFA (28.5%), C16:1 (1.20%), C18:1 (26.5%), C20:1 (0.21%), total n-6 PUFA (35.1%), total n-3 PUFA (3.47%), C20:5 (eicosapentanoic acid, EPA) (0.72%), C22:6 (docosapentanoic acid, DPA) (0.39%) and C22:5 (docosahexanoic acid, DHA) (2.04%) to determine the effect of specific genotypes in subjects with similar plasma FA concentrations based on habitual diet.

**Table 3:** Significant associations and interactions for baseline plasma FA (%FA) and *APOE* genotype on metabolic variables in the LIPGENE Dietary Fatty Acid Intervention Study (n = 416).

	*E2* carriers		*E3/E3*		*E4* carriers		*P *		
	Low FA	High FA	Low FA	High FA	Low FA	High FA	FA	Genotype	FA × Genotype
C16:0	(n = 21)	(n = 25)	(n = 137)	(n = 122)	(n = 53)	(n = 58)			
HOMAIR	2.21 ± 0.26	2.98 ± 0.40	2.51 ± 0.13	2.78 ± 0.15	2.35 ± 0.26^a^	3.41 ± 0.26^b^	< 0.001	0.747	0.018
Insulin (mmol/L)	8.18 ± 0.82	10.9 ± 1.41	9.61 ± 0.48	10.4 ± 0.53	8.70 ± 0.89^a^	12.2 ± 0.87^b^	0.001	0.641	0.033
N-6 PUFA	(n = 25)	(n = 21)	(n = 123)	(n = 126)	(n = 59)	(n = 52)			
Apo E (mg/L)	65.8 ± 7.52^a,c^	42.3 ± 3.04^b^	41.9 ± 1.17^a,d^	38.0 ± 0.88^b^	41.1 ± 1.97^a,d^	34.7 ± 1.91^b^	< 0.001	< 0.001	0.012
TRL-C (mmol/L)	0.63 ± 0.08^a,c^	0.25 ± 0.03^b^	0.43 ± 0.02^a,d^	0.25 ± 0.01^b^	0.58 ± 0.06^a,d^	0.32 ± 0.03^b^	< 0.001	0.003	0.029
C20:1	(n = 20)	(n = 26)	(n = 128)	(n = 131)	(n = 58)	(n = 53)			
Apo E (mg/L)	69.6 ± 9.46^a,c^	44.1 ± 2.28^b^	42.1 ± 1.16^d^	37.6 ± 0.86	38.5 ± 1.93^d^	37.6 ± 2.1	0.001	< 0.001	0.004
N-3 PUFA	(n = 20)	(n = 26)	(n = 136)	(n = 123)	(n = 60)	(n = 51)			
Apo CIII (mg/L)	190 ± 17.7^a^	154 ± 11.4^b^	156 ± 4.11	158 ± 4.40	158 ± 7.91	159 ± 5.73	0.005	0.458	0.022
Apo E (mg/L)	63.6 ± 9.44^a,c^	48.6 ± 3.62^b,c^	39.9 ± 1.01^d^	39.8 ± 1.07	39.4 ± 2.12^d^	36.3 ± 1.67^d^	0.001	< 0.001	0.037
NEFA (µmol/L)	585 ± 53.2	745 ± 46.2	596 ± 21.4	595 ± 18.2	674 ± 34.9	600 ± 28.4	0.200	0.148	0.042
EPA/C20:5 (n-3)	(n = 20)	(n = 26)	(n = 136)	(n = 123)	(n = 50)	(n = 61)			
Apo CIII (mg/L)	182 ± 17.9^a^	160 ± 11.8^b^	153 ± 3.74	162 ± 4.80	157 ± 8.71	160 ± 5.95	0.001	0.365	0.041
Apo E (mg/L)	66.9 ± 9.2^a,c^	46.1 ± 3.5^b^	40.2 ± 1.1^d^	39.5 ± 1.0	40.4 ± 2.5^d^	36.1 ± 1.5	< 0.001	< 0.001	0.002
DHA/C22:6 (n-3)	(n = 20)	(n = 26)	(n = 122)	(n = 137)	(n = 65)	(n = 46)			
Apo E (mg/L)	55.1 ± 6.4^c^	55.1 ± 6.4	48.0 ± 0.9^d^	41.5 ± 1.1	39.5 ± 2.0^d^	35.9 ± 1.8	0.029	< 0.001	0.020

Abbreviations: DHA, docosahexanoic acid; EPA, eicosapentanoic acid; FA, fatty acid; HOMA-IR, homeostasis model assessment of insulin resistance; NEFA, non-esterified fatty acid; PUFA, polyunsaturated fatty acid TRL-C, triglyceride rich lipoprotein cholesterol fraction. Values are means ± s.e.m. Data were analyzed using general linear models with adjustment for age, baseline BMI, baseline alcohol intake (g/d), sex, exercise level index, center and smoking status. Where P for plasma FA × genotype <0.05, a post hoc Bonferroni test used to determine between group effects (low FA, less than median plasma FA; high FA, greater than median plasma FA). Superscript letters ^a^ and ^b^ denote significant differences between low and high FA within each genotype; letters ^c^ and ^d^ significant differences between genotypes within each FA group, P < 0.05.

At baseline, significant interactions between *APOE* and plasma n-6 PUFA were observed for TRL-C. Whilst all three genotype groups had significantly higher TRL-C concentrations in the low plasma n-6 PUFA group, compared with the high plasma n-6 PUFA group, *E2* carriers with low plasma n-6 had significantly higher TRL-C than *E3/E3* and *E4* carriers.

**Figure 1:**
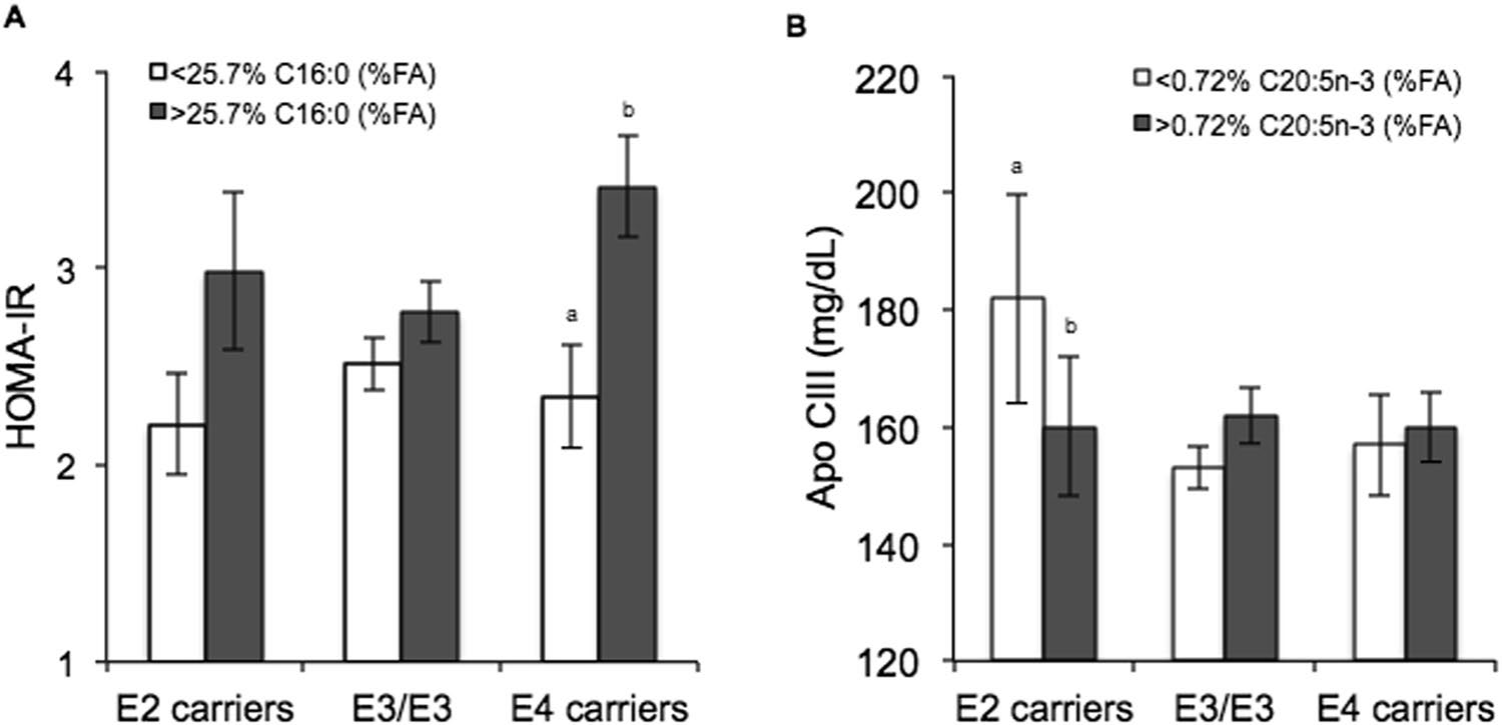
Effect of the *APOE* genotype and </> median baseline plasma fatty acid concentration on (**A**) HOMA-IR (*APOE* × C16:0 interaction, *P* = 0.018) and (**B**) apo CIII (*APOE* × C20:5n-3 interaction, *P* = 0.041) in metabolic syndrome subjects. Values are means ± s.e.m. Letters (a and b) are used to denote significant differences between plasma fatty acid groups within the same genotype, *P* < 0.05 using post hoc Bonferroni.

The *APOE* genotype also influenced apo E and apo CIII concentrations according to plasma n-6 PUFA and n-3 PUFA, and plasma n-3 PUFA respectively. Lower plasma n-3 PUFA, n-6 PUFA, EPA and C20:1 was associated with significantly higher apo E concentrations in *E2* carriers. The same interaction was observed with the *E3/E3* genotype for n-6 PUFA. In the low plasma n-3 PUFA, n-6 PUFA, EPA, DHA and C20:1 groups, *E2* carriers had significantly higher concentrations of apo E than *E3/E3* and *E4* carriers. For apo CIII, dichotomization according to median FA revealed significantly lower concentrations of apo CIII with a higher proportion plasma total n-3 PUFA and EPA in *E2* carriers only (Fig. 1B).

Another key finding in the present study was the variation in insulin resistance according to *APOE* genotype and plasma total SFA; nutrient-gene interactions were observed for both HOMA-IR and insulin. FA analysis revealed that *E4* carriers with higher proportions of plasma C16:0 had significantly higher HOMA-IR and insulin than those with lower plasma C16:0 (Fig. 1A). Interaction between C18:0 and *APOE* on HOMA-IR and insulin approached significance.

Nutrient-gene interactions were observed between total n-3 PUFA and *APOE* for plasma NEFA and plasma SFA and *APOE* on CRP (data not shown); however, no specific differences between genotypes were detected after post-hoc analysis.

### Plasma FA and genotype effects following FA dietary intervention

Significant gene-nutrient interactions between *APOE* and change in plasma SFA, PUFA and LC n-3 PUFA, evaluated as a continuous variable (as a % of total FA), on metabolic biomarkers were only observed for LC n-3 PUFA (Table 4). An increase in LC n-3 PUFA was represented by a >0% change in LC n-3 PUFA whereas a decrease in LC n-3 PUFA was represented by a <0% change in LC n-3 PUFA (categorical data). Plasma FA’s changed in all subjects.

**Table 4:** Effects of significant change in plasma LC n-3 PUFA (%FA) and *APOE* genotype interactions on metabolic variables following the LIPGENE Dietary Fatty Acid Intervention Study (*n* = 351).

	*E2* carriers		*E3/E3*		*E4* carriers		*P*		
	Decrease LC n-3 (n = 15)	Increase LC n-3 (n = 26)	Decrease LC n-3 (n = 75)	Increase LC n-3 (n = 142)	Decrease LC n-3 (n = 29)	Increase LC n-3 (n = 64)	FA	Genotype	FA × Genotype
TRL-C (mmol/L)	−0.05 ± 0.05	0.09 ± 0.09^c^	0.03 ± 0.02	−0.02 ± 0.02^d^	0.07 ± 0.04	−0.02 ± 0.04	0.561	0.077	0.021
Apo CII (mg/L)	−6.37 ± 3.08^a,c^	1.19 ± 2.41^b^	1.69 ± 1.32	−1.45 ± 0.72	3.13 ± 2.07^d^	−0.15 ± 1.21	0.259	0.112	0.002
Apo E (mg/L)	−3.95 ± 1.52	1.58 ± 2.34^c^	0.52 ± 0.93	−1.97 ± 0.77^d^	0.03 ± 1.68	−1.41 ± 1.47^d^	0.228	0.161	0.025

Abbreviations: FA, fatty acid; LC n-3 PUFA, long-chain omega-3 polyunsaturated fatty acid (comprising EPA and DHA); decrease LC n-3, less than 0% change in plasma LC n-3 PUFA; increase LC n-3, greater than 0% change in plasma LC n-3 PUFA; TRL-C, triglyceride rich lipoprotein cholesterol fraction. Values are means ± s.e.m. Data were analyzed using general linear models with adjustment for age, sex, center, change in weight (week 12 – baseline) and the respective pre-intervention variable. Where P for plasma FA × genotype <0.05, a post hoc Bonferroni test used to determine between group effects. Superscript letters ^a^ and ^b^ denote significant differences between low and high FA within each genotype; letters ^c^ and ^d^ significant differences between genotypes within each FA group (P < 0.05).

Following dietary FA intervention, significant interactions were observed between *APOE* genotype and change in LC n-3 PUFA for TRL-C. Whereas an increase in the proportion of plasma LC n-3 PUFA was associated with an increase in TRL-C concentrations in *E2* carriers, a reduction in TRL-C was observed in the *E3/E3* genotype.

**Figure 2:**
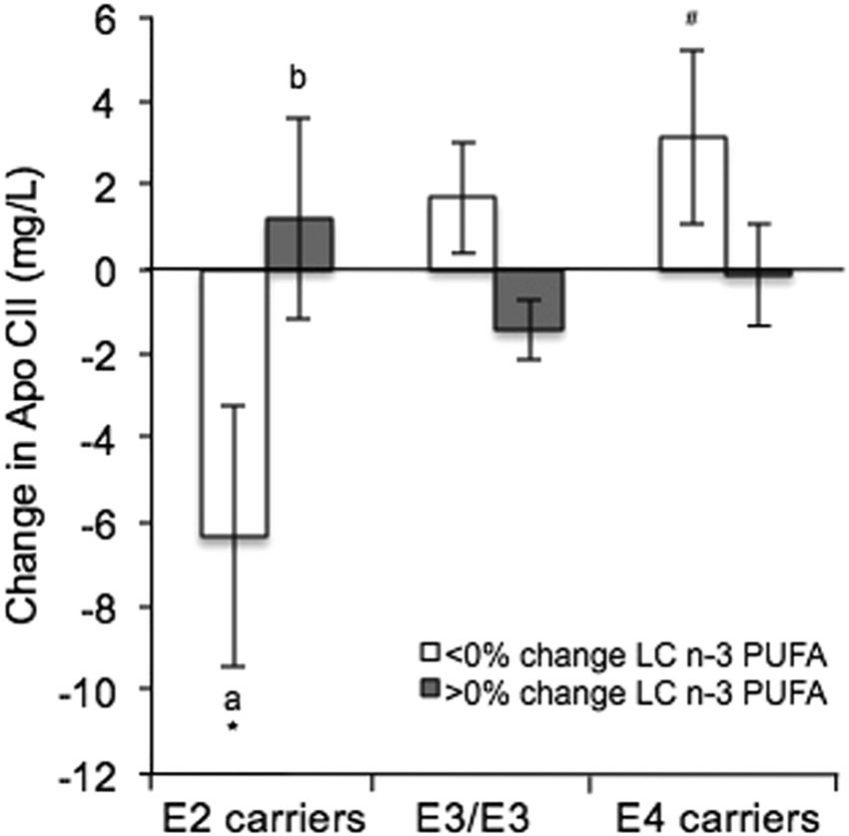
Effect of the *APOE* genotype and change in plasma LC n-3 PUFA concentration on change in apo CII (*APOE* × LC n-3 PUFA interaction, *P* = 0.041) following the LIPGENE Dietary Fatty Acid Intervention in metabolic syndrome subjects. Values are means ± s.e.m. Letters (a and b) are used to denote significant differences between plasma fatty acid groups within the same genotype; symbols (* and ^#^) are used to denote significant differences between genotypes within the same fatty acid group, *P* < 0.05 using post hoc Bonferroni.

The *APOE* genotype also influenced the apo CII and apo E response to change in LC n-3 PUFA. Apo CII concentrations reduced in *E2* carriers following a reduction in plasma LC n-3 PUFA, however, they increased when plasma LC n-3 PUFA was raised. In *E4* carriers, a significantly higher apo CII concentration was observed in response to a reduction in the proportion of plasma LC n-3 PUFA than in *E2* carriers (Fig. 2). For apo E, *E2* carriers displayed an increase in apo E concentration following an increase in plasma LC n-3 PUFA, whereas reductions were observed in *E3/E3* and *E4* carriers.

## Discussion

A beneficial impact of increasing plasma LC n-3 PUFA on apo CII concentrations in *E2* carriers, and a detrimental association between high plasma SFA and insulin resistance in *E4* carriers at baseline was observed. To our knowledge, this is the first study to examine the impact of change in plasma FA, from dietary fat intervention, on lipids and insulin resistance in a MetS population.

The allele distribution in the LIPGENE cohort was similar to that reported in previous Caucasian populations^35^, as was the geographical cline observed for greater frequency of the ε4 allele and lower plasma apo E concentrations in Northern Europeans compared with Southern Europeans^36–38^. In the European Atherosclerosis Research Study, a “clear-cut” gradient for ε4 allele frequency which followed the coronary heart disease (CHD) mortality gradient was reported^38^. The 10-year incidence of CHD is 2.68 times greater in Northern than in Southern Europeans^39^ and it has been proposed that the differential effects of the ε4 and ε2 alleles may contribute to this difference in risk across Europe^40^. In a recent systematic review, Khan *et al*. reported a positive association between *APOE* genotype and TC (*P* trend: 2 × 10^−152^), with highest TC in *E4* carriers, which support our observations from the LIPGENE cohort (*E*2 < *E*3*/E*3 < *E*4)^41^. Circulating LDL-C and apo B concentrations were also significantly greater in *E4* carriers at baseline compared with *E2* carriers, as observed previously in MetS populations^14^. However, there were no differences in baseline plasma FA or dietary fat intake.

In terms of receptor affinity it is now recognized that there are no major differences between the E3 and E4 protein isoform binding to the LDL-receptor (LDL-R)^42^, yet *E4* subjects are often found to have higher TC and LDL-C. A possible explanation for this anomaly is a preferential association of the E4 protein with TRL particles (as opposed to HDL in *E3/E3*)^43^. This is proposed to result in more apo E per TRL particle in *E4* carriers which increases competition at the LDL-R^44^, resulting in less uptake of LDL and increased circulating plasma cholesterol. Additionally, VLDL remnant lipolytic conversion to LDL is reported to be at a faster rate in *E4* subjects^43^. Impaired recycling of TRL-derived apoE4 has also been associated with cholesterol accumulation^45^. In contrast the LDL-R binding affinity of the E2 isoform is less than 2% of the strength of E3 and E4 resulting in slower clearance of VLDL and dietary derived chylomicron (CM) remnants from the blood and typically lower TC and LDL-C concentrations^46, 47^. The decreased rate of LDL formation and subsequent up-regulation of LDL-R in *E2* carriers results in increased circulating TAG^35^.

At baseline, there were no significant differences in TRL-C between genotypes, however a significant nutrient-gene interaction was observed for plasma n-6 PUFA, with *E2* carriers having significantly higher TRL-C at lower intakes of n-6 PUFA than *E3/E3* and *E4* carriers. In addition to serum TAG, *E2* carriers have been reported to have higher concentrations of circulating TRL-C compared with the *E3/E3* wild-type^19^, as was observed in the LIPGENE cohort. A possible explanation of this is the slower CM remnant and VLDL clearance in *E2* carriers. In the present study, higher plasma n-6 PUFA was associated with lower TRL-C concentrations, in all genotype groups. This is suggestive of a beneficial impact of plasma n-6 PUFA on plasma cholesterol concentrations. Whilst an interaction was observed between change in LC n-3 PUFA and *APOE* genotype for TRL-C (following FA dietary intervention), significant differences between groups were not identified. Previous analysis of the LIPGENE data found a beneficial impact of dietary LC n-3 PUFA on LDL density^48^.

Up to 30% of the variability in serum apo E concentrations has been attributed to the *APOE* polymorphism, with highest concentrations in *E2* carriers and the lowest in *E4* carriers, however concentrations are also influenced by sex, age and geography^37, 49^. Apo E is located on HDL-C, CM and intermediate-density lipoproteins (IDL) and binds to the LDL-R. Due to the impaired binding affinity of APOE2 protein, there is reduced uptake of CM and IDL (compared with E3 and E4), resulting in higher circulating apo E concentrations. In the present study, *E2* carriers had significantly higher plasma apo E concentrations when compared with both *APOE3* and *E4* carriers. Our data confirms that the *APOE* locus is a major determinant of the plasma apo E concentration even in the presence of MetS. Kypreos *et al*. reported that plasma apo E has anti-inflammatory properties^50^ which, in addition to lower TC concentration, may contribute towards the 20% decreased risk of CHD observed in *E2* carriers. At baseline, high plasma total n-3 and n-6 PUFA levels were associated with lower concentration of apo E in *E2* carriers. However, an increase in LC n-3 PUFA following the LIPGENE dietary FA intervention raised apo E concentrations in *E2* carriers, compared with a reduction observed in *E4* carriers and *E3/E3*.

Apo CIII concentrations were significantly lower in *E2* individuals with high concentrations of plasma total n-3 PUFA, n-6 PUFA and EPA. Apo CIII is an inhibitor of lipoprotein lipase (LPL) and TRL catabolism^51^; thus, increased concentrations will promote increased circulating TAG. The TAG lowering effect of LC n-3 PUFA is well documented^26^, and may be associated with increased LPL activity and gene expression and/or a reduction in apo CIII^52^. Given that elevated TAG is an independent risk factor for CVD^53^, these findings suggest a benefit of increased plasma n-3 PUFA in *E2* carriers, particularly as this group often present with elevated plasma TAG. When exposed to a lower proportion of plasma n-3 PUFA/ EPA, *E2* carriers had significantly more apo CIII than *E4* carriers. Whereas interactions between *PPARα* and *APOC-III* genotypes and LC n-3 PUFA have been shown to modulate apo CIII concentrations^54, 55^, the interaction with *APOE* is novel. Following the LIPGENE dietary FA intervention a significant *APOE* × LC n-3 PUFA was observed for apo CII. In contrast to apo CIII, apo CII activates LPL and increases TAG catabolism. In *E2* carriers, an increase in plasma LC n-3 PUFA was associated with an increase in apo CII concentrations, compared with a decrease when LC n-3 PUFA intake reduced. This provides further evidence for a beneficial impact of LC n-3 PUFA in *E2* carriers in relation to TAG metabolism.

An interesting finding in the present analysis was the detrimental association between high plasma C16:0 on markers of insulin resistance, defined by HOMA-IR > 2.6^56^, in *E4* carriers. Although *E4* carriers with low plasma C16:0 at baseline were not ‘insulin resistant’ (HOMA-IR, =2.35), those with high plasma C16:0 had a 31% greater HOMA-IR. This represents a novel finding and may suggest that *E4* carriers with MetS are particularly sensitive to the detrimental metabolic effects of high palmitic acid (C16:0) levels. It is of note that increased plasma C16:0 has been associated with risk of type 2 diabetes, which could be in part due to increased insulin resistance^57^; our findings indicate that this relationship is amplified in *E4* carriers. Previous studies have also shown a negative impact of diets rich in C16:0 and SFA on insulin sensitivity index (S_I_) in overweight individuals^58, 59^. However, several studies including the LIPGENE study, found no impact of reducing SFA on S_I_^29^. The lack of effect found in the primary LIPGENE analysis highlights the importance of the *APOE* genotype (E4) on insulin-glucose homeostasis in metabolically challenged individuals.

At baseline in the current study, there were no genotype-dependent differences on insulin resistance, supporting previous reports by Ragogna *et al*.^60^. The frequency of *APOE* genotypes were also compared across HOMA-IR quintiles in the Framingham cohort, with no genotypic association reported^61^. However, others reported a significant genotype-obesity interaction for glucose and insulin, whereby obese *E4* men (BMI ≥ 30 kg/m^2^) had higher fasted glucose and insulin compared to both non-obese men and non apoE4 obese subjects^62^. It has been suggested that the APOE-obesity interaction may intensify insulin resistance in men. A potential mechanism for this is accelerated lipid peroxidation due to increased TC and LDL-C, and reduced LDL diameter in obese *E4* carriers^62^.

Evaluation of the interactions between *APOE* genotype and FA on metabolic markers was not the primary aim of LIPGENE; thus, the use of a retrospective genotyping approach was unavoidable and could be seen as a potential limitation. Inevitably this resulted in uneven group sizes, although reasonable total numbers were available in each genotype group and adequate study power was achieved. Based on the observed group size and differences in LDL-C between *E2* and *E4*, and *E3/E3* and *E4* at baseline, the powers (1-β) achieved were 0.99 (effect size = 0.87, critical t = 1.97, P = 0.05) and 0.76 (effect size = 0.30, critical t = 1.97, P = 0.05) respectively. The power achieved for differences in HOMAIR between low and high plasma C16:0 in *E4* carriers was 0.82 (effect size = 0.55, critical t = 1.98, P = 0.05) and for differences in apo CII between E2 and E4 carriers following LC n-3 PUFA reduction the power achieved was 0.72 (effect size = 0.83, critical t = 2.02, P = 0.05). A strength of the present analysis was the use of plasma FA to explore nutrient-gene interactions in the *APOE* gene; plasma FA concentrations indicate the specific exposure of cells to FA and provide insight into potential mechanisms, whereas dietary intake is prone to misreporting.

Plasma SFA, MUFA and PUFA can be informative with respect to dietary FA intakes and positive correlations have been observed between reported dietary total PUFA and LC n-3 PUFA intake (%TE) and corresponding FA in blood lipid fractions^63^. However, correlations between dietary and plasma SFA and MUFA are weak^63^. Given that plasma FA can also be synthesized *de novo* from endogenous fatty acids and non-fatty acid sources, associations found between plasma SFA and measured outcomes may not only reflect dietary intake of FA. A potential limitation of this analysis is that subject groupings may alter for each FA investigated such that the effects of a given FA may be confounded by the presence of higher or lower quantities of other FA. However, this method does enable us to evaluate the impact of changes in plasma FA on concomitant changes in outcome measures. Furthermore, analysis irrespective of diet group allocation increases the sample size and statistical power, and reduces the confounding influence of non-adherence to dietary intervention.

In conclusion, we observed that a high proportion of plasma palmitic acid was associated with greater insulin resistance in *E4* carriers with MetS, whereas increasing plasma LC n-3 PUFA beneficially increased apo CII and reduced apo CIII concentrations in *E2* carriers, which may confer TAG lowering benefits. These interactions represent novel findings, which should be explored further. In the context of personalized nutrition, our data suggest that individuals with MetS may benefit from personalized dietary interventions based on *APOE* genotype.

## Electronic supplementary material

Supplementary Information - APOE genotype in the Metabolic Syndrome
