# Supplementary material for: APOE genotype influences insulin resistance, apolipoprotein CII and CIII according to plasma fatty acid profile in the Metabolic Syndrome
Source: Sci Rep. 2017 Jul 24;7:6274. doi: 10.1038/s41598-017-05802-2 (PMC5524844; doi:10.1038/s41598-017-05802-2)
Supplement: Supplementary file 1 — Supplementary Information - APOE genotype in the Metabolic Syndrome [file 41598_2017_5802_MOESM1_ESM.doc]

**TITLE:**

***APOE* genotype influences insulin resistance, apolipoprotein CII and CIII according to plasma fatty acid profile in the Metabolic Syndrome**

**AUTHORS:**

Rosalind Fallaize, Andrew L. Carvalho-Wells, Audrey C. Tierney, Carmen Marin, Beata Kieć-Wilk, Aldona Dembińska-Kieć, Christian A. Drevon, Catherine DeFoort, José Lopez-Miranda, Ulf Risérus, Wim H Saris, Ellen E. Blaak, Helen M. Roche and Julie A. Lovegrove

**CORRESPONDING AUTHOR:**

Professor Julie A. Lovegrove, Hugh Sinclair Unit of Human Nutrition, Department of Food and Nutritional Sciences, University of Reading, Whiteknights, Reading RG6 6AP, United Kingdom. Tel: +44 (0) 118 378 6418; Fax: +44 (0) 118 931 0080; Email: [j.a.lovegrove@reading.ac.uk](mailto:j.a.lovegrove@reading.ac.uk)

**Supplementary Table 1.** Classes, subclasses and individual plasma fatty acids (FA) evaluated in the LIPGENE dietary fatty acid intervention study

| **Fatty acid** | **Lipid components** |
| --- | --- |
| Total SFA | C14:0, C16:0, C18:0 |
| Myristic acid | C14:0 |
| Palmitic acid | C16:0 |
| Stearic acid | C18:0 |
| Total MUFA | C16:1, C18:1, C20:1 |
| Palmitoleic acid | C16:1 |
| Oleic acid | C18:1 |
| Eicosenoic acid | C20:1 |
| n-6 PUFA | C18:2, C18:3, C20:3, C20:4, C22:4 |
| Linoleic | C18:2 |
| Gamma-linolenic acid | C18:3 |
| Dihomo-gamma-linolenic acid | C20:3 |
| Arachidonic acid | C20:4 |
| Adrenic acid | C22:4 |
| n-3 PUFA | C18:4, C20:5, C22:5, C22:6 |
| Stearidonic acid | C18:4 |
| Eicosapentanoic acid (EPA)* | C20:5 |
| Docosapentaenoic acid | C22:5 |
| Docosahexanoic acid (DHA)* | C22:6 |

Abbreviations: FA, fatty acid; MUFA, monounsaturated fatty acid; PUFA, polyunsaturated fatty acid; SFA, saturated fatty acid. * denotes FA comprising LC n-3 PUFA, long-chain omega-3 polyunsaturated fatty acid.

**Supplementary Table 2.** Baseline plasma fatty acids according to *APOE* genotype in the LIPGENE Dietary Fatty Acid Intervention Study (*n=*442; males *n=*194, females *n=*248)

|  | *All (n=442)* | *E2 carriers (n=46)* | *E3/E3 (n=264)* | *E4 carriers (n=115)* | *E2/E4 (n=17)* | *P* |
| --- | --- | --- | --- | --- | --- | --- |
| C14:0 | 1.94 ± 0.05 | 2.09 ± 0.17 | 1.82 ± 0.06 | 2.19 ± 0.10 | 1.93 ± 0.16 | 0.217 |
| C16:0 | 26.8 ± 0.25 | 27.2 ± 0.76 | 26.5 ± 0.32 | 27.2 ± 0.51 | 26.9 ± 0.58 | 0.588 |
| C18:0 | 4.22 ± 0.10 | 4.14 ± 0.36 | 4.32 ± 0.13 | 4.10 ± 0.20 | 3.62 ± 0.38 | 0.652 |
| C16:1 | 1.43 ± 0.05 | 1.39 ± 0.16 | 1.31 ± 0.06 | 1.74 ± 0.12 | 1.35 ± 0.21 | 0.143 |
| C18:1 | 26.4 ± 0.21 | 27.4 ± 0.75 | 26.3 ± 0.27 | 26.6 ± 0.43 | 26.2 ± 0.83 | 0.083 |
| C20:1 | 0.21 ± 0.011 | 0.25 ± 0.04 | 0.22 ± 0.02 | 0.19 ± 0.01 | 0.20 ± 0.04 | 0.588 |
| C18:4 | 0.036 ± 0.002 | 0.044 ± 0.008 | 0.036 ± 0.007 | 0.035 ± 0.004 | 0.031 ± 0.007 | 0.413 |
| C20:5 | 0.95 ± 0.04 | 0.91 ± 0.12 | 0.94 ± 0.06 | 1.00 ± 0.08 | 1.01 ± 0.20 | 0.671 |
| C22:5 | 0.46 ± 0.02 | 0.54 ± 0.06 | 0.44 ± 0.02 | 0.48 ± 0.04 | 0.48 ± 0.08 | 0.542 |
| C22:6 | 2.16 ± 0.06 | 2.17 ± 0.17 | 2.24 ± 0.08 | 1.97 ± 0.11 | 2.34 ± 0.36 | 0.252 |
| C18:2 | 27.3 ± 0.26 | 26.1 ± 0.71 | 27.6 ± 0.34 | 26.9 ± 0.54 | 28.7 ± 1.24 | 0.192 |
| C18:3 | 0.063 ± 0.003 | 0.051 ± 0.008 | 0.066 ± 0.004 | 0.062 ± 0.006 | 0.030 ± 0.007 | 0.088 |
| C20:3 | 0.85 ± 0.02 | 0.77 ± 0.06 | 0.90 ± 0.03 | 0.80 ± 0.04 | 0.77 ± 0.10 | 0.067 |
| C20:4 | 6.82 ± 0.12 | 6.60 ± 0.32 | 7.04 ± 0.16 | 6.49 ± 0.22 | 6.29 ± 0.54 | 0.525 |
| C22:4 | 0.18 ± 0.01 | 0.22 ± 0.03 | 0.17 ± 0.01 | 0.19 ± 0.02 | 0.13 ± 0.02 | 0.346 |

Values are means ± s.e.m, plasma fatty acids are presented as a % of total. Models were adjusted for center, gender, age and BMI. Where *P* for genotype < 0.05, a post-hoc Bonferroni test were used to determine a between group effect.

**Supplementary Table 3.** Baseline dietary fat intake according to APOE genotype in the LIPGENE Dietary Fatty Acid Intervention Study (n=412; males n=184, females n=228).

Values are means ± s.e.m. MUFA, monounsaturated fat; PUFA, polyunsaturated fat. Models were adjusted for center, gender, age and BMI. Where *P* for genotype < 0.05, a post-hoc Bonferroni test were used to determine a between group effect. Superscript numbers denote reduced sample sizes due to missing data: ^1^ all, n=292; *E2 carriers*, n=28; *E3/E3*, n=172, *E4 carriers*, n=80; *E2/E4,* n=12 and ^2^ all, n=182; *E2 carriers*, n=21; *E3/E3*, n=105, *E4 carriers*, n=50; *E2/E4,* n=6.

|  | *All (n=412)* | *E2 carriers (n=43)* | *E3/E3 (n=244)* | *E4 carriers (n=108)* | *E2/E4 (n=17)* | *P* |
| --- | --- | --- | --- | --- | --- | --- |
| Total fat (g) | 85.4 ± 1.6 | 86.1 ± 5.2 | 87.8 ± 2.1 | 79.4 ± 2.7 | 89.3 ± 6.8 | 0.607 |
| Saturated fat (g) | 29.6 ± 0.6 | 31.0 ± 2.3 | 29.5 ± 0.8 | 28.9 ± 1.2 | 31.5 ± 2.5 | 0.637 |
| MUFA g) | 31.9 ± 0.7 | 31.6 ± 2.2 | 33.6 ± 0.9 | 27.8 ± 1.1 | 33.2 ± 3.5 | 0.607 |
| PUFA (g) | 13.2 ± 0.3 | 13.3 ± 1.0 | 13.5 ± 0.5 | 12.5 ± 0.6 | 13.0 ± 1.3 | 0.431 |
| N-6 PUFA (g)^1^ | 9.2 ± 0.3 | 9.2 ± 0.9 | 9.1 ± 0.5 | 9.3 ± 0.6 | 10.5 ± 1.4 | 0.276 |
| N-3 PUFA (g)^1^ | 1.47 ± 0.07 | 1.61 ± 0.21 | 1.42 ± 0.10 | 1.46 ± 0.11 | 1.95 ± 0.40 | 0.586 |
| Trans fat (g)^2^ | 2.44 ±0.15 | 2.89 ± 0.48 | 2.55 ± 0.21 | 1.95 ± 0.19 | 2.94 ± 1.43 | 0.277 |
